# Anemia in disadvantaged children aged under five years; quality of care in primary practice

**DOI:** 10.1186/s12887-019-1543-2

**Published:** 2019-06-04

**Authors:** Casey Mitchinson, Natalie Strobel, Daniel McAullay, Kimberley McAuley, Ross Bailie, Karen M. Edmond

**Affiliations:** 1 Perth Children’s Hospital, Child and Adolescent Health Service, Government of Western Australia, Perth, Western Australia Australia; 2 Medical School, The University of Western Australia, Perth, Western Australia Australia; 3 University Centre for Rural Health, The University of Sydney, Lismore, New South Wales Australia

**Keywords:** Child, Anemia, Primary care

## Abstract

**Background:**

Anemia rates are over 60% in disadvantaged children yet there is little information about the quality of anemia care for disadvantaged children.

**Methods:**

Our primary objective was to assess the burden and quality of anemia care for disadvantaged children and to determine how this varied by age and geographic location. We implemented a cross-sectional study using clinical audit data from 2287 Indigenous children aged 6–59 months attending 109 primary health care centers between 2012 and 2014. Data were analysed using multivariable regression models.

**Results:**

Children aged 6–11 months (164, 41.9%) were less likely to receive anemia care than children aged 12–59 months (963, 56.5%) (adjusted odds ratio [aOR] 0.48, CI 0.35, 0.65). Proportion of children receiving anemia care ranged from 10.2% (92) (advice about ‘food security’) to 72.8% (728) (nutrition advice). 70.2% of children had a hemoglobin measurement in the last 12 months. Non-remote area families (115, 38.2) were less likely to receive anemia care compared to remote families (1012, 56.4%) (aOR 0.34, CI 0.15, 0.74). 57% (111) aged 6–11 months were diagnosed with anemia compared to 42.8% (163) aged 12–23 months and 22.4% (201) aged 24–59 months. 49% (48.5%, 219) of children with anemia received follow up.

**Conclusions:**

The burden of anemia and quality of care for disadvantaged Indigenous children was concerning across all remote and urban locations assessed in this study. Improved services are needed for children aged 6–11 months, who are particularly at risk.

## Background

Anemia is a major public health issue globally with an estimated prevalence of 47% in children aged under 5 years. [1] Prevalence is reported to be 70% in children living in low income countries and over 30% in disadvantaged Indigenous children aged under 5 years worldwide. [2, 3] Children are born with high hemoglobin concentrations but levels drop after 6 months of age due to depletion of iron stores with the most vulnerable period between 6 and 11 months. [4–7] Iron deficiency is the most important cause of anemia. [8] However, the cycle of poverty, poor environmental conditions, chronic infection, malabsorption and anorexia affecting disadvantaged children and families is also well recognised. [9, 10] Iron deficiency and other forms of anemia are associated with long term deficits in cognitive development and poor educational outcomes, especially in the youngest infants. [11, 12]

Reducing anemia rates requires complex and long-standing changes to nutritional intake, education levels, economic status and the social determinants of health. [9, 10] However, primary health care services have an important role in prevention, early detection and treatment. In Australia, the national government advises primary care providers to administer a ‘child health check’ annually to each Indigenous child across the country. [13] These ‘checks’ are standardised, based on best practice guidelines and include measurements for growth and screening for hemoglobin at least once per year for high risk groups, as well as breastfeeding promotion, dietary and complementary feeding advice, discussion of housing and food security and recommendations about social support services. [13]

Yet, there is little information about how well anemia services are being implemented in busy primary care settings, especially those in remote areas, which service highly disadvantaged communities. Also, despite the high burden, to our knowledge only one study has assessed anemia burden and the quality of anemia care provided to infants aged 6–11 months. [14]

The Audit for Best Practice in Chronic Disease (ABCD) continuous quality improvement (CQI) program was developed for Australian Indigenous primary health care centers for the prevention and management of chronic disease. [15–17] The ABCD program aims to improve service delivery using plan-do-study-act (PDSA) cycles (including analysing current practice, implementing change and then encouraging service providers to assess the impact of the change). [15]

Our primary objective was to assess the quality of anemia care provided to disadvantaged children attending the ABCD primary care centers and to determine how this varied by age. Secondary objectives were to assess the effect of geographic location and to describe the burden of anemia (hemoglobin < 110 g/dl) in children aged 6–59 months.

## Methods

### Study setting

This was a cross sectional study using audit data from children aged 6–59 months from 109 Indigenous primary health care centers in remote, rural and urban areas across Australia between 2012 and 2014. Details of these methods are published elsewhere. [18, 19] The characteristics of the primary care clinics and health care providers are presented in Table 1.

**Table 1:** Key characteristics by age and geographic location in Indigenous children aged 6–59 months

	Total	Age (months)			Geographic location		
		6–11	12–23	24–59	Remote	Rural	Urban
Total	2287	430 (18.8%)	532 (23.3%)	1325 (57.9%)	1861 (81.4%)	346 (15.1%)	80 (3.5%)
Health Service Characteristics							
Governance							
Aboriginal community controlled health service	528 (23.1%)	88 (20.5%)	118 (22.2%)	322 (24.3%)	293 (15.7%)	208 (60.1%)	27 (33.8%)
Government health service	1759 (76.9%)	342 (79.5%)	414 (77.8%)	1003 (75.7%)	1568 (84.3%)	138 (39.9%)	53 (66.3%)
Health service provider who first saw the child							
Indigenous health worker	318 (13.9%)	49 (11.4%)	67 (12.6%)	202 (15.2%)	205 (11%)	91 (26.3%)	22 (27.5%)
Nurse	1584 (69.3%)	321 (74.7%)	381 (71.6%)	882 (66.6%)	1385 (74.4%)	159 (46%)	40 (50%)
GP	259 (11.3%)	50 (11.6%)	60 (11.3%)	149 (11.3%)	157 (8.4%)	85 (24.6%)	17 (21.3%)
Other	109 (4.8%)	8 (1.9%)	20 (3.8%)	81 (6.1%)	98 (5.3%)	10 (2.9%)	1 (1.3%)
Missing	17 (0.7%)	2 (0.5%)	4 (0.8%)	11 (0.8%)	16 (0.9%)	1 (0.3%)	0 (0%)
Year of data collection							
2012	448 (19.6%)	87 (20.2%)	107 (20.1%)	254 (19.2%)	284 (15.3%)	144 (41.6%)	20 (25%)
2013	1251 (54.7%)	230 (53.5%)	276 (51.9%)	745 (56.2%)	1095 (58.8%)	156 (45.1%)	0 (0%)
2014	588 (25.7%)	113 (26.3%)	149 (28%)	326 (24.6%)	482 (25.9%)	46 (13.3%)	60 (75%)
Population size							
≤ 500	816 (35.7%)	105 (24.4%)	196 (36.8%)	515 (38.9%)	802 (43.1%)	14 (4.0%)	0 (0%)
501–999	458 (20%)	73 (17%)	101 (19%)	284 (21.4%)	410 (22%)	39 (11.3%)	9 (11.3%)
≥ 1000	1013 (44.3%)	252 (58.6%)	235 (44.2%)	526 (39.7%)	649 (34.9%)	293 (84.7%)	71 (88.8%)
Child characteristics							
Sex of child							
Male	1156 (50.5%)	217 (50.7%)	272 (51.1%)	667 (50.3%)	941 (50.6%)	175 (50.6%)	40 (50%)
Female	1131 (49.5%)	213 (49.5%)	260 (48.9%)	658 (49.7%)	920 (49.4%)	171 (49.4%)	40 (50%)
Type of child health check completed in the last 12 months							
MBS 715	928 (40.6%)	175 (40.7%)	229 (43%)	524 (39.6%)	928 (40.6%)	781 (42%)	117 (33.8%)
Other child health check	587 (25.7%)	120 (27.9%)	147 (27.6%)	320 (24.2%)	587 (25.7%)	462 (24.8%)	111 (32.1%)
Not known / not recorded	772 (33.8%)	135 (31.4%)	156 (29.3%)	481 (36.3%)	772 (33.8%)	618 (33.2%)	118 (34.1%)
Reason for last clinic attendance							
Acute care	1145 (50.1%)	210 (48.8%)	271 (50.9%)	664 (50.1%)	1145 (50.1%)	945 (50.8%)	163 (47.1%)
Immunisation	324 (14.2%)	80 (18.6%)	87 (16.4%)	157 (11.8%)	324 (14.2%)	233 (12.5%)	73 (21.1%)
Child health check	515 (22.52%)	93 (21.63%)	112 (21.05%)	310 (23.4%)	515 (22.52%)	418 (22.46%)	80 (23.12%)
Other	303 (13.2%)	47 (10.9%)	62 (11.7%)	194 (14.6%)	303 (13.2%)	265 (14.2%)	30 (8.7%)

*CQI* Continuous Quality Improvement

### Clinic procedures

The annual child health checks were implemented by trained accredited nurses using standardised equipment (including Hemocue™ hemoglobinometers, electronic weighing scales and stadiometers) that were regularly calibrated according to the manufacturer’s instructions. Annual weight measurements, height measurements and blood samples (heel [6–11 months] or finger prick [≥12 months] were taken from each child using standard operating procedures and calibrated hemoglobinometers. [20] Formal laboratory full blood examinations (FBE) using venous samples were only taken when there was specific concerns about a child. The annual child health checks also included advice about breastfeeding and healthy foods, treatment of abnormal hemoglobin measurements, assessment of oral health, assessment of developmental milestones and discussion about social and emotional needs. [13, 21–26]

### Data collection

Audits of medical records were performed annually by participating primary health care centers. Records were eligible for inclusion if they were from children i) aged 6–59 months at the time of the audit; ii) resident in the community for 6 months or more (for children aged < 12 months resident for at least 50% of time since birth); and iii) with no major health problem such as congenital abnormalities. If a center had 30 or less eligible children, all records were audited. In larger centers 30 files were randomly selected. Children were excluded if they had not attended the clinic in the preceding 12 months.

A standardised audit tool was used to collect data from selected clinic records. Child characteristics included: birth date, age, sex, Indigenous status, attendance at the clinic in the last 12 months, reason for most recent attendance and provision of any type of child health screening in the previous 12 months. Heath center characteristics included governance (Aboriginal Community Controlled Health Service or government health service), location (urban, rural, remote), population catchment area, and the number of CQI audits the primary care center had completed.

The audit tool included five coded items that related to the quality of anemia care. The auditors scored ‘yes’ if there had been any description in the client file in the previous 12 months of: (i) advice about breastfeeding, (ii) nutrition advice to the mother or child about healthy foods and the minimum acceptable diet, (iii) advice about food security (discussion including availability, affordability, accessibility and attainment and storage of appropriate and nutritious foods on a regular and reliable basis), (iv) hemoglobin measurement, (v) follow up for children with anemia including nutrition advice, iron treatment and repeat hemoglobin measurements within 2 months. Items were ‘not applicable’ if they were not specified in the guidelines for children of that age in the particular state or territory. [13]

### Definitions

A composite measure of ‘quality of anemia care’ was defined as documentation in the child’s file of the two items required for all children aged 6–59 months (i) the child’s caregiver had received nutrition advice about healthy foods and the minimum acceptable diet and (ii) the child had received a hemoglobin measurement in the past 12 months. The composite measure was scored as ‘yes’ if both areas were documented in the client file.

A child was defined as having ‘abnormal hemoglobin levels’ according to the clinical practice guidelines in their state or territory for a child of that age (hemoglobin cut point of 100, 105 or 110 g/dl). ‘Anemia’ was defined according to the World Health Organization guidelines as a hemoglobin level less than 110 g/dl for children aged 6–59 months. [27]

Geographic location was defined using categories from the Accessibility/Remoteness Index of Australia (ARIA). [28] The ARIA index was developed by the Commonwealth Department of Health and Aged Care to define remoteness based on accessibility/road distances to service centers. The index includes five categories ranging from 1 (Highly accessible) to 5 (Very remote). In this study ‘urban’ was defined as ARIA category 1, ‘rural’ included ARIA categories 2–4 and ‘remote’ was ARIA category 5.

### Statistical analysis

The primary outcome measure was the proportion of children who received the composite measure of anemia care. Our primary objective was to compare the proportion of children who received the composite measure of anaemia care who were aged 6–11 months with children who were aged 12–59 months.

We calculated that a sample size of 2000 children in our study provided 90% power to detect a difference of at least 10% in the quality of anemia care between those aged 6–11 months and 12–59 months. This calculation assumed a 5% significance level, a baseline quality of care of 50% in those 6–11 months of age and a ratio of 1:2 for those aged 6–11 months and 12–59 months of age.

Unadjusted and adjusted odds ratios (ORs) with 95% confidence intervals (95% CI) were calculated to assess the association between key characteristics, including age (6–11, 12–23, 24–59 months), geographic location and delivery of anemia care. Multilevel binomial generalised estimating equation models with an exchangeable correlation structure and robust standard errors were used with primary care center as the clustering variable. To adjust for potential confounding, multivariable regression models were constructed a priori which included variables: age, sex of child, geographic location, governance structure, CQI participation and year of data collection. Data analyses were conducted using STATA 13.1.

## Results

### General characteristics

**Fig. 1:**
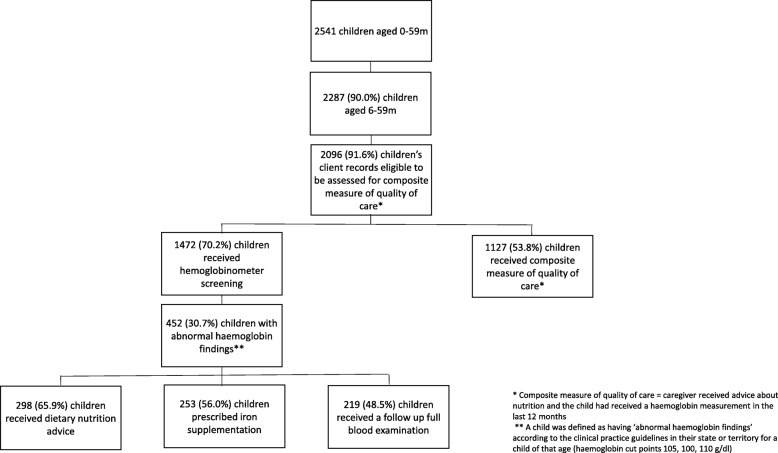
Participant flow chart

Our study included audits of clinical records for 2287 Indigenous children aged 6 to 59 months who visited one of 109 primary health care centers across Australia during 2012 to 2014, inclusive. Nineteen percent (430) of audits were for children 6 to 11 months of age, 23% (532) 12 to 23 months of age and 58% (1325) 24 to 59 months of age (Table 1, Fig. 1). Health service and child characteristics were similar between different age groups (Table 1). Only 3 % (80) of children were from urban centers whilst over 80% (1861) were from remote areas and 15% (346) from rural areas (Table 1).

The audit of clinical records showed that our composite measure of quality of anemia care was completed in 54% (1127) of children (Table 2). The proportion of families with a record of receiving specific services ranged from 76% (728) of families who were reported to be educated about breastfeeding to only 10% (92) for advice about food security.

**Table 2:** Anaemia care by age and geographic location in Indigenous children aged 6–59 months

	Eligible primary care centres n (%)	Number of client records assessed^a^ n (%)	Proportion receiving care n (%)	Age (months)			Geographic location		
				6–11 n (%)	12–23 n (%)	24–59 n (%)	Remote	Rural	Urban
Total	109 (100%)	2287	2287	430 (18.8%)	532 (23.3%)	1325 (57.9%)	1861 (81.4%)	346 (15.1%)	80 (3.5%)
Anaemia care									
Anticipatory guidance									
Breastfeeding (< 2 years)	109 (100%)	962 (42.1%)	728 (75.7%)	376 (87.4%)	352 (66.2%)	N/A	638 (80.6%)	64 (53.8%)	26 (51%)
Nutrition advice	109 (100%)	2287 (100%)	1665 (72.8%)	344 (80%)	434 (81.6%)	887 (66.9%)	1373 (73.8%)	233 (67.3%)	59 (73.8%)
Food security	109 (100%)	899 (39.3%)	92 (10.2%)	29 (15.9%)	29 (13.9%)	34 (6.7%)	66 (9.4%)	20 (11.4%)	6 (25%)
Child health surveillance									
Haemoglobin documented in last 12 months	109 (100%)	2096 (91.6%)	1472 (70.2%)	195 (49.9%)	381 (76.5%)	896 (74.2%)	1343 (74.8%)	109 (41.8%)	20 (50%)
Follow up of abnormal findings^b^									
Dietary/nutrition advice	109 (100%)	452 (19.8%)	298 (65.9%)	51 (65.4%)	115 (71%)	132 (62.3%)	258 (63.5%)	34 (85%)	6 (100%)
Prescription of iron supplement	109 (100%)	452 (19.8%)	253 (56.0%)	40 (51.3%)	97 (59.9%)	116 (54.7%)	239 (58.9%)	11 (27.5%)	3 (50%)
Follow-up FBE or haemoglobin within 2 months	109 (100%)	452 (19.8%)	219 (48.5%)	40 (51.3%)	76 (46.9%)	103 (48.6%)	208 (51.2%)	7 (17.5%)	4 (66.7%)
Composite measure of quality of care^c^	109 (100%)	2096 (91.6%)	1127 (53.8%)	164 (41.9%)	322 (64.7%)	641 (53.1%)	1012 (56.4%)	95 (36.4%)	20 (50%)

*CQI* Continuous Quality Improvement, *FBE* Full blood examination

^a^Proportions are less than 100% if the service is not included in the best practice guidelines for children of that age

^b^A child was defined as having ‘abnormal haemoglobin findings’ according to the clinical practice guidelines in their state or territory for a child of that age (haemoglobin cut points 105, 100, 110 g/dl)

^c^Caregiver received advice about nutrition and the child had received a haemoglobin measurement in the last 12 months

### Age and geographic location

There was a strong association between anemia care and age group. Children aged 6 to 11 months (164, 41.9%) were 52% less likely to receive the composite measure compared to those aged 12–59 months (963, 56.5%) (Tables 2 and 3). Children aged 6 to 11 months (195, 49.9%) were also 71% less likely to receive a hemoglobin screening measurement compared to those aged 12–59 months (1277, 74.9%) (Tables 2 and 3).

**Table 3:** Association between key characteristics and anaemia care in Indigenous children aged 6–59 months

	Total number	Number that received composite measure	OR (95% CI)	*P* value	aOR^a^ (95% CI)	*P* value
Total	2096	1127 (53.8%)				
Health service characteristics						
Geographic location						
Remote	1795	1012 (56.4%)	1.00		1.00	
Non remote	301	115 (38.2%)	0.30 (0.14, 0.62)	0.001	0.34 (0.15, 0.74)	0.006
CQI participation (number of audits completed)						
1	349	149 (42.7%)	1.00		1.00	
2	523	253 (48.4%)	1.49 (0.63, 3.50)	0.360	1.06 (0.45, 2.50)	0.899
≥ 3	1224	725 (59.2%)	2.27 (1.07, 4.83)	0.033	1.71 (0.81, 3.62)	0.161
Governance						
Aboriginal community controlled health service	388	194 (50%)	0.81 (0.44, 1.51)	0.511	1.06 (0.56, 2.03)	0.856
Government health service	1708	933 (54.6%)	1.00		1.00	
Health service provider who first saw the child						
Indigenous health worker	255	120 (47.1%)	0.83 (0.65, 1.07)	0.158	0.85 (0.65, 1.10)	0.220
Nurse	1500	822 (54.8%)	1.00		1.00	
General practitioner	225	118 (52.4%)	1.05 (0.76, 1.45)	0.770	1.11 (0.80, 1.54)	0.519
Other	99	56(56.6%)	1.02 (0.66, 1.57)	0.943	1.01 (0.65, 1.56)	0.964
Missing	17	11 (64.7%)				
Year of data collection						
2012	418	210 (50.2%)	1.00		1.00	
2013	1114	577 (51.8%)	0.99 (0.51, 1.92)	0.976	0.68 (0.37, 1.25)	0.218
2014	564	340 (60.3%)	1.33 (0.64, 2.77)	0.445	1.20 (0.62, 2.32)	0.599
Population size						
≤ 500	782	390 (49.9%)	1.00		1.00	
501–999	420	250 (59.5%)	1.41 (0.79, 2.52)	0.243	2.27 (1.22, 4.26)	0.010
≥ 1000	894	487 (54.5%)	1.20 (0.74, 1.96)	0.464	2.02 (1.27, 3.20)	0.003
Child characteristics						
Age of child						
6-11 m	391	164 (41.9%)	0.57 (0.41, 0.79)	0.001	0.55 (0.39, 0.78)	0.001
12-23 m	498	322 (64.7%)	1.60 (1.30, 1.97)	< 0.001	1.63 (1.31, 2.03)	< 0.001
24-59 m	1207	641 (53.1%)	1.00		1.00	
Sex of child						
Male	1055	573 (54.3%)	1.00		1.00	
Female	1041	554 (53.2%)	0.98 (0.85, 1.13)	0.777	0.98 (0.85, 1.14)	0.827
Reason for last clinic attendance						
Acute care	1052	553 (52.6%)	0.66 (0.55, 0.79)	< 0.001	0.65 (0.54, 0.78)	< 0.001
Immunisation	296	119 (40.2%)	0.63 (0.51, 0.79)	< 0.001	0.63 (0.50, 0.79)	< 0.001
Child health check	464	275 (59.3%)	1.00		1.00	
Other	284	180 (63.4%)	0.83 (0.63, 1.11)	0.206	0.81 (0.61, 1.08)	0.153

*OR* Odds ratio, *aOR* Adjusted odds ratio

^a^Adjusted for age, sex, year of data collection, geographic location, governance, CQI participation

The quality of anemia care was strongly associated with location of the health care center. Children attending clinics in non-remote areas (115, 38.2%) were 66% less likely to receive the composite measure compared to those from remote areas (1012, 56.4%) (aOR 0.34, CI 0.15, 0.74) (Tables 2 and 3).

### Abnormal findings

The proportion of children who had a hemoglobin measurement within the preceding 12 months and who had abnormal findings (Hb < 100–110 g/dl) was 30.7% (452) (Table 4). Abnormal findings were higher in children aged 6–11 months (78, 40.0%) compared with those 12–59 months of age (374, 29.3%) (Table 4). Children attending clinics in non-remote areas (46, 35.7%) had a similar prevalence of abnormal findings compared to those from remote regions (406, 30.2%) (Table 4).

**Table 4:** Associations between key characteristics and abnormal findings in Indigenous children aged 6–59 months

	Total number	Evidence of anaemia				
	n	n (%)	OR (95% CI)	*P* value	aOR (95% CI)^a^	*P* value
Total	1472	452 (30.7%)				
Child characteristics						
Age of child						
6-11 m	195	78 (40.0%)	2.28 (1.62, 3.21)	< 0.001	2.36 (1.65, 3.39)	< 0.001
12-23 m	381	162 (42.5%)	2.62 (2.04, 3.35)	< 0.001	2.64 (2.07, 3.38)	< 0.001
24-59 m	896	212 (23.7%)	1.00		1.00	
Sex of child						
Male	748	225 (30.1%)	1.00		1.00	
Female	724	227 (31.4%)	0.99 (0.79, 1.25)	0.949	1.01 (0.79, 1.28)	0.955
Reason for last clinic attendance						
Acute care	750	236 (31.5%)	1.13 (0.86, 1.50)	0.380	1.13 (0.84, 1.53)	0.405
Immunisation	140	39 (27.9%)	0.93 (0.57, 1.52)	0.773	0.87 (0.52, 1.44)	0.590
Child health check	341	87 (25.5%)	1.00		1.00	
Other	241	90 (37.3%)	1.29 (0.86, 1.95)	0.223	1.42 (0.92, 2.20)	0.114
Health service characteristics						
Geographic location						
Remote	1343	406 (30.2%)	1.00		1.00	
Non remote	129	46 (35.7%)	1.29 (0.68, 2.45)	0.435	0.90 (0.38, 2.12)	0.805
Number of audit rounds completed						
1	215	82 (38.1%)	1.00		1.00	
2	400	145 (36.3%)	0.93 (0.50, 1.75)	0.832	0.80 (0.44, 1.44)	0.448
≥ 3	857	225 (26.3%)	0.54 (0.31, 0.93)	0.026	0.47 (0.28, 0.78)	0.003
Governance						
Aboriginal community controlled health service	233	92 (39.5%)	1.58 (1.03, 2.44)	0.037	1.68 (1.00, 2.83)	0.052
Government health service	1239	360 (29.1%)	1.00		1.00	
Health service provider who first saw the child						
Indigenous health worker	158	39 (24.7%)	0.78 (0.55, 1.10)	0.158	0.80 (0.57, 1.13)	0.201
Nurse	1087	344 (31.6%)	1.00		1.00	
General practitioner	133	38 (28.6%)	1.03 (0.71, 1.50)	0.858	0.96 (0.64, 1.43)	0.833
Other	82	28 (34.1%)	1.19 (0.71, 1.97)	0.511	1.39 (0.80, 2.40)	0.239
Missing	12	3 (25.0%)				
Year of data collection						
2012	253	85 (33.6%)	1.00		1.00	
2013	763	193 (25.3%)	0.65 (0.40, 1.07)	0.091	0.73 (0.43, 1.25)	0.255
2014	456	174 (38.2%)	1.29 (0.77, 2.18)	0.337	1.33 (0.76, 2.34)	0.315
Population size						
≤ 500	564	145 (25.7%)	1.00		1.00	
501–999	306	96 (31.4%)	1.31 (0.74, 2.32)	0.348	1.57 (0.83, 2.95)	0.165
≥ 1000	602	211 (35.1%)	1.56 (1.02, 2.38)	0.040	1.54 (1.02, 2.33)	0.041

*OR* Odds ratio, *aOR* Adjusted odds ratio

^a^Adjusted for age, sex, year of data collection, geographic location, governance, CQI participation

Treatment and follow up of children diagnosed with abnormal Hb levels was low. Only 65.9% (298) of children with abnormal Hb levels received dietary and nutrition advice, 56.0% (253) were prescribed an iron supplement and 48.5% (219) had a follow-up hemoglobin within 2 months (Table 2). The rates of treatment and follow-up appeared similar between different age groups and geographic regions (remote versus non-remote) (Table 2).

32.2% (475) children were defined as having ‘anemia’ according to WHO criteria (Hb less than 110 g/dl). Levels were lower in younger children (56.9% children aged 6–11 months, 42.8% children aged 12–23 months and 22.4% children aged 24–59 months) (Table 5). Levels were similar in remote and non remote children. Only one child aged 6–11 months had ‘severe anemia’ (Hb < 70 g/dl).

**Table 5:** Haemoglobin levels by age group and geographic location in children aged 6–59 months

		Total number	Mean (sd) Hb (g/dl)	Median (IQR) Hb (g/dl)	Range (min-max) Hb (g/dl)	Proportion with Hb < 70 g/dl n (%)	Proportion with Hb < 100 g/dl n (%)	Proportion with Hb < 110 g/dl n (%)
Total		1472	113.1 (11.1)	114 (107–120)	61–158	1 (0.1%)	163 (11.1%)	475 (32.3%)
6–11 months		195	107.8 (12.0)	108 (100–115)	61–158	1 (0.01%)	45 (23.1%)	111 (56.9%)
	Remote	184	107.8 (11.7)	108 (100–115)	75–135	0 (0.01%)	43 (23.4%)	106 (57.6%)
	Non-remote	11	107.4 (17.6)	111 (102–119)	61–124	1 (0.01%)	2 (18.2%)	5 (45.5%)
12–23 months		381	109.5 (11.8)	111 (102–117)	70–148	0 (0%)	73 (19.2%)	163 (42.8%)
	Remote	344	109.4 (11.8)	111 (102–117)	70–148	0 (0%)	66 (19.2%)	149 (43.3%)
	Non-remote	37	110.4 (11.8)	112 (105–119)	79–129	0 (0%)	7 (18.9%)	14 (37.8%)
24–59 months		896	115.7 (9.7)	115 (110–122)	83–147	0 (0%)	45 (5.0%)	201 (22.4%)
	Remote	815	115.9 (9.6)	115 (110–122)	87–147	0 (0%)	39 (4.8%)	172 (21.1%)
	Non-remote	81	113.0 (10.2)	113 (107–120)	83–136	0 (0%)	6 (7.4%)	29 (35.8%)

*Hb* haemoglobin

*Sd* standard deviation

*IQR* interquartile range

## Discussion

To our knowledge, this is the largest published study describing the quality of anemia care provided to disadvantaged children in primary health care centers. Anemia prevalence was 33% overall and 57% in children aged 6–11 months. Yet only 54% of children received the composite measure of anemia care, 76% of caregivers received nutrition advice, 70% of children had a hemoglobin measurement within the preceding 12 months and only 48% received follow up care for anemia. Young children aged 6–11 months had the poorest quality of care despite having the highest anemia rates. Health centres in remote areas appeared recorded better quality of care than non-remote areas.

The prevalence of anaemia that we reported in our study was similar to anaemia prevalence reported for other disadvantaged children in low and middle income countries globally (especially east and southeast Asia [29%] and southern Africa [30%]). [2] Our rates were also similar to disadvantaged children in high income countries including Inuit children (36%) in Canada [3], urban African-American children (25–35%) in the US [29, 30] and Native Alaskan infants (35%). [31] Rural risk factors for anemia are well known and include tropical diseases and severe food insecurity. However, in both urban and rural areas, poor education levels and poverty also limit food purchasing and the provision of an adequate nutritional intake. [32, 33]

We also found the highest anemia prevalence in children aged 6–11 months (57%) and 12–23 months (43%). Infant anemia is well known to be due to poor maternal nutrition, poor complementary food intake and gastro intestinal infections. [34, 35] Low birth weight and maternal anemia are also important determinants of early onset anemia, [11, 36].

We also reported concerningly low quality of anemia care (54%). Provision of nutrition advice and screening to families by primary care providers in Australia was reported to be as low as 20% in 2011. [14] However, many efforts have been made to improve primary and secondary prevention of anemia in remote areas including training of health care providers, CQI initiatives and community consultation. [37] The quality of anemia care we report this study is substantially better than reported in the 2011 study. These findings are encouraging given the ongoing challenges of high staff turnover and difficulty in accessing professional development in remote areas.

We found six other studies that reported on the poor quality of anemia care for disadvantaged children, [4, 14, 38–42] Of these, one assessed quality of care in infants aged 6 months and compared to older age groups.(38)In our study children aged 6–11 months had the highest anemia burden (56.9%) but were two fold less likely to receive anemia care compared to those aged 12–59 months.

Interestingly, the quality of health centre care in urban areas was significantly poorer than remote areas in our study. This may be due to some participation bias by health centres. i.e., participation was voluntary and more ‘better quality’ health centres may have volunteered in remote than urban areas.. Other possible reasons include difficulties in locating children who live in crowded urban environments, lack of funding for urban based care from local and national governments and lack of community health workers or other ancillary staff to help with communication and follow up. [43] This can result in fragmentation of care with many children receiving care from multiple different service providers. Similar findings have been reported from other high-income urban environments including studies of type 2 diabetes [44], immunisation, [45] and adult preventative health care services. [46]

We also reported poor anemia treatment and follow up in the disadvantaged children in our study. Our low follow up rate (49%) may be explained by the frequent migration between city and country locations commonly seen in disadvantaged families. [14, 47] However, we also reported that only 66% of children with anemia received dietary/nutrition advice and 56% were prescribed an iron supplement at the time of diagnosis. These findings are most likely explained by high staff turnover and the need for ongoing staff trainings. Our standard operating procedures state that nutritional advice and iron therapy should commence immediately while waiting for laboratory results. We have now conducted refresher training in both remote and urban areas and are continuing close follow up of these concerning findings. We are also focusing on ‘structures of care’ such as education and training, capacity building and improvements in the organisation of health systems. [14, 48]

Long term neurodevelopmental and educational outcomes have been linked to early deprivation and micronutrient intake, [11, 12] so it is concerning that very young infants aged 6–11 months had both high levels of anemia and poor quality of care and follow-up in our study. This low prevalence of care for our youngest and most vulnerable infants may be because of the perception that anemia commences later in childhood. [14]

There were some limitations to our study. Some items may not have been documented in client records thus there may have been under reporting of the level of care provided. Participation of health services was voluntary therefore limiting generalisability. We were unable to collect data on cause of anemia e.g. iron deficiency so we cannot comment on aetiology specific issues. We are also aware that our anemia rates were reported only in the 70% of children who received screening for anemia. Children that did not receive screening may be more disadvantaged and have even higher anemia burden. Our anemia burden data relied on capillary samples (heel prick and finger prick) analysed by hemoglobinometers. Venous blood full blood examinations (FBE) are well known to be the gold standard technique for measuring hemoglobin levels. However, there have been many hemoglobinometer diagnostic accuracy studies that report high levels of sensitivity, specificity and level of agreement with venous Coulter samples if the hemoglobinometer is used by well trained staff under optimal situations such as in our study. [49, 50]

Strengths of our study included the large sample size and multicenter design which included a large number of primary health care centers across different regions of Australia. Within the statistical analysis we controlled for confounders such as age, sex, year, geographic location, governance and CQI participation and we feel that residual confounding was unlikely. We also controlled for the effects of clustering of health care centers.

## Conclusion

Anemia continues to be an important issue for disadvantaged children in urban, rural and remote areas. In our study children aged 6–11 months had the highest anemia rates but the poorest quality of care. Improving care for these vulnerable children is especially needed. This includes improved training and capacity building of primary care providers in the care of young children, the delivery of standardised health checks and ensuring appropriate follow up and treatment.

## Data Availability

The datasets generated and/or analysed during the current study are not publicly available due to the lack of an online platform but are available from the corresponding author on reasonable request.

## Abbreviations

ABCD: Audit for Best Practice in Chronic Disease
aOR: adjusted odds ratio
CI: Confidence interval
CQI: Continuous quality improvement
FBE: Full blood examinations
OR: Odds ratio
PDSA: Plan-do-study-act

## References

[1] McLean E, Cogswell M, Egli I, Wojdyla D, de Benoist B (2009). Worldwide prevalence of anaemia, WHO vitamin and mineral nutrition information system, 1993-2005. Public Health Nutr.

[2] Stevens GA, Finucane MM, De-Regil LM, Paciorek CJ, Flaxman SR, Branca F, Pena-Rosas JP, Bhutta ZA, Ezzati M (2013). Nutrition impact model study G. global, regional, and national trends in haemoglobin concentration and prevalence of total and severe anaemia in children and pregnant and non-pregnant women for 1995-2011: a systematic analysis of population-representative data. Lancet Glob Health.

[3] Christofides A, Schauer C, Zlotkin SH (2005). Iron deficiency and anemia prevalence and associated etiologic risk factors in first nations and Inuit communities in northern Ontario and Nunavut. Can J Public Health.

[4] Sears David, Mpimbaza Arthur, Kigozi Ruth, Sserwanga Asadu, Chang Michelle A., Kapella Bryan K., Yoon Steven, Kamya Moses R., Dorsey Grant, Ruel Theodore (2015). Quality of Inpatient Pediatric Case Management for Four Leading Causes of Child Mortality at Six Government-Run Ugandan Hospitals. PLOS ONE.

[5] Ewusie JE, Ahiadeke C, Beyene J, Hamid JS (2014). Prevalence of anemia among under-5 children in the Ghanaian population: estimates from the Ghana demographic and health survey. BMC Public Health.

[6] Austin AM, Fawzi W, Hill AG (2012). Anaemia among Egyptian children between 2000 and 2005: trends and predictors. Matern Child Nutr.

[7] Xin QQ, Chen BW, Yin DL, Xiao F, Li RL, Yin T, Yang HM, Zheng XG, Wang LH (2017). Prevalence of Anemia and its risk factors among children under 36 months old in China. J Trop Pediatr.

[8] Lopez A, Cacoub P, Macdougall IC, Peyrin-Biroulet L (2016). Iron deficiency anaemia. Lancet.

[9] Khambalia AZ, Aimone AM, Zlotkin SH (2011). Burden of anemia among indigenous populations. Nutr Rev.

[10] Marmot M (2011). Social determinants and the health of indigenous Australians. Med J Aust.

[11] Balarajan Y, Ramakrishnan U, Ozaltin E, Shankar AH, Subramanian SV (2011). Anaemia in low-income and middle-income countries. Lancet.

[12] Grantham-McGregor S, Ani C (2001). A review of studies on the effect of iron deficiency on cognitive development in children. J Nutr.

[13] Department of Health. Health assessment for Aboriginal and Torres Strait Islander people (MBS Item 715). Medicare Benefits Schedule - Note AN.0.44. Canberra: Commonwealth of Australia. http://www9.health.gov.au/mbs/fullDisplay.cfm?type=note&qt=NoteID&q=AN.0.44 Accessed 9 Apr 2016

[14] Bar-Zeev SJ, Kruske SG, Barclay LM, Bar-Zeev N, Kildea SV (2013). Adherence to management guidelines for growth faltering and anaemia in remote dwelling Australian aboriginal infants and barriers to health service delivery. BMC Health Serv Res.

[15] Bailie RS, Si D, O'Donoghue L, Dowden M (2007). Indigenous health: effective and sustainable health services through continuous quality improvement. Med J Aust.

[16] Bailie R, Si D, Connors C, Weeramanthri T, Clark L, Dowden M, O'Donohue L, Condon J, Thompson S, Clelland N (2008). Study protocol: audit and best practice for chronic disease extension (ABCDE) project. BMC Health Serv Res.

[17] Bailie R, Si D, Shannon C, Semmens J, Rowley K, Scrimgeour DJ, Nagel T, Anderson I, Connors C, Weeramanthri T (2010). Study protocol: national research partnership to improve primary health care performance and outcomes for indigenous peoples. BMC Health Serv Res.

[18] Edmond KM, McAuley K, McAullay D, Matthews V, Strobel N, Marriott R, Bailie R (2018). Quality of social and emotional wellbeing services for families of young indigenous children attending primary care centers; a cross sectional analysis. BMC Health Serv Res.

[19] McAullay D, McAuley K, Bailie R, Mathews V, Jacoby P, Gardner K, Sibthorpe B, Strobel N, Edmond K (2018). Sustained participation in annual continuous quality improvement activities improves quality of care for aboriginal and Torres Strait islander children. J Paediatr Child Health.

[20] Department of Health. Heel prick blood collection. In: Child and Adolescent Community Health, ed. Western Australia: Child and Adolescent Health Service, Department of Health; 2016.

[21] NACCHO/RACGP (2012). National guide to a preventive health assessment for Aboriginal and Torres Strait Islander people.

[22] Kimberley Aboriginal Medical Services Council (KAMSC), WA Country Health Service (WACHS) Kimberley (2011). Healthy Kids.

[23] Kimberley Aboriginal Medical Services Council (KAMSC), WA Country Health Service (WACHS) Kimberley (2015). Anaemia in children.

[24] Queensland Health. Internet: https://www.health.qld.gov.au/rrcsu/html/health-check-forms Accessed 20 April 2016.

[25] Queensland Health (2016). Royal Flying Doctor Service (Queensland section). Primary clinical care manual 9th edition.

[26] Remote Primary Health Care Manuals (2017). CARPA standard treatment manual.

[27] WHO (2011). Haemoglobin concentrations for the diagnosis of anaemia and assessment of severity. Vitamin and Mineral Nutrition Information System.

[28] Commonwealth Department of Health and Aged Care. Measuring remoteness: Accessibility/remoteness index of Australia (ARIA). Revised edition: Occasional Papers: New Series Number 14; 2001. Canberra.

[29] Bogen DL, Krause JP, Serwint JR (2001). Outcome of children identified as anemic by routine screening in an inner-city clinic. Arch Pediatr Adolesc Med.

[30] Bogen DL, Duggan AK, Dover GJ, Wilson MH (2000). Screening for iron deficiency anemia by dietary history in a high-risk population. Pediatrics.

[31] Gessner BD (2009). Geographic and racial patterns of anemia prevalence among low-income Alaskan children and pregnant or postpartum women limit potential etiologies. J Pediatr Gastroenterol Nutr.

[32] Gracey M, King M (2009). Indigenous health part 1: determinants and disease patterns. Lancet.

[33] ABS (2015). Food security.

[34] Hipgrave DB, Fu X, Zhou H, Jin Y, Wang X, Chang S, Scherpbier RW, Wang Y, Guo S (2014). Poor complementary feeding practices and high anaemia prevalence among infants and young children in rural central and western China. Eur J Clin Nutr.

[35] Wirth JP, Rohner F, Woodruff BA, Chiwile F, Yankson H, Koroma AS, Russel F, Sesay F, Dominguez E, Petry N (2016). Anemia, micronutrient deficiencies, and malaria in children and women in Sierra Leone prior to the Ebola outbreak - findings of a cross-sectional study. PLoS One.

[36] Ayoya Mohamed Ag, Ngnie-Teta Ismael, Séraphin Marie Nancy, Mamadoultaibou Aissa, Boldon Ellen, Saint-Fleur Jean Ernst, Koo Leslie, Bernard Samuel (2013). Prevalence and Risk Factors of Anemia among Children 6–59 Months Old in Haiti. Anemia.

[37] Australian Institute of Health and Welfare (2016). Australia’s health 2016. Australia’s health series no. 15. Cat. no. AUS 199.

[38] Bailie RS, Si D, Dowden MC, Connors CM, O'Donoghue L, Liddle HE, Kennedy CM, Cox RJ, Burke HP, Thompson SC (2008). Delivery of child health services in indigenous communities: implications for the federal government's emergency intervention in the Northern Territory. Med J Aust.

[39] McLennan JDS, M. (2016). Anemia screening and treatment outcomes of children in a low-resource Community in the Dominican Republic. J Trop Pediatr.

[40] Crowell R, Pierce MB, Ferris AM, Slivka H, Joyce P, Bernstein BA, Russell-Curtis S (2005). Managing anemia in low-income toddlers: barriers, challenges and context in primary care. J Health Care Poor Underserved.

[41] Kempe A, Beaty B, Englund BP, Roark RJ, Hester N, Steiner JF (2000). Quality of care and use of the medical home in a state-funded capitated primary care plan for low-income children. Pediatrics.

[42] Biondich PG, Downs SM, Carroll AE, Laskey AL, Liu GC, Rosenman M, Wang J, Swigonski NL (2006). Shortcomings in infant iron deficiency screening methods. Pediatrics.

[43] Ware V. Improving access to urban and regional early childhood services. Resource sheet no. 17. Produced for the closing the gap clearinghouse. Cnberra: Australian Institute of Health and Welfare, Melbourne: Australian Institute of Family Studies,, 2012.

[44] Matthews V, Schierhout G, McBroom J, Connors C, Kennedy C, Kwedza R, Larkins S, Moore E, Thompson S, Scrimgeour D (2014). Duration of participation in continuous quality improvement: a key factor explaining improved delivery of type 2 diabetes services. BMC Health Serv Res.

[45] O'Grady KA, Krause V, Andrews R (2009). Immunisation coverage in Australian indigenous children: time to move the goal posts. Vaccine.

[46] Bailie RS, Si D, Connors CM, Kwedza R, O'Donoghue L, Kennedy C, Cox R, Liddle H, Hains J, Dowden MC (2011). Variation in quality of preventive care for well adults in indigenous community health centres in Australia. BMC Health Serv Res.

[47] Aquino D, Marley J, Senior K, Leonard D, Joshua A, Huddleston A, Ferguson H, Helmer J, Hadgraft N, Hobson V (2013). Early childhood nutrition and anaemia prevention project Darwin: The Fred Hollows Foundation, Indigenous Australia Program.

[48] Strobel Natalie A., McAuley Kimberley, Matthews Veronica, Richardson Alice, Agostino Jason, Bailie Ross, Edmond Karen M., McAullay Daniel (2018). Understanding the structure and processes of primary health care for young indigenous children. Journal of Primary Health Care.

[49] Cohen AR, Seidl-Friedman J (1988). HemoCue system for hemoglobin measurement. Evaluation in anemic and nonanemic children. Am J Clin Pathol.

[50] Mills AF, Meadows N (1989). Screening for anaemia: evaluation of a haemoglobinometer. Arch Dis Child.

